# Progress of CT aortic angiography combined with coronary artery in the evaluation of acute aortic syndrome

**DOI:** 10.3389/fcvm.2022.1036982

**Published:** 2022-11-21

**Authors:** Hengbin An, Ruigang Xie, Yinghui Ge, Tianyun Wang

**Affiliations:** ^1^School of Basic Medicine, Xinxiang Medical University, Xinxiang, China; ^2^Fuwai Central China Cardiovascular Hospital, Zhengzhou, China; ^3^Henan Provincial Key Laboratory of Cardiology Medical Imaging, Zhengzhou, China

**Keywords:** acute aortic syndrome, CT angiography, multi-slice spiral CT, contrast agent, coronary heart disease

## Abstract

Acute aortic syndrome (AAS) is a group of cardiovascular diseases that seriously threaten human life and health. AAS patients are often complicated with coronary artery disease and other related diseases, which require rapid and clear clinical diagnosis to avoid serious adverse events. In recent years, with the progress of science and technology, a variety of computer tomography (CT) angiography techniques have been applied in the clinic, and the diagnosis rate of AAS with coronary heart disease (CAD) has greatly increased. At the same time, the development of surgical technology and endovascular repair technology has significantly reduced the mortality and complication rate of AAS surgery. In the clinical diagnosis of AAS and related diseases, CT aortic angiography (CTA) combined with coronary CTA is increasingly applied to identify related diseases. Here, the current research progress on the technique of aortic CTA combined with coronary CTA is reviewed.

## Introduction

**FIGURE 1:**
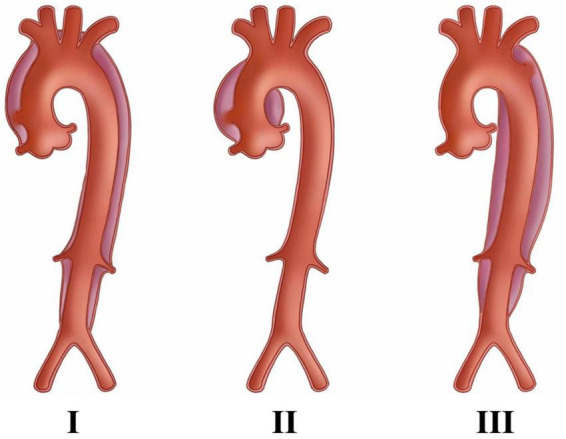
The DeBaKey classification of aortic dissection, in which Type I and Type II required attention to coronary artery condition and requires surgery, whereas Type III is better suited for percutaneous stent placement. The picture is from Lin et al. (13).

Acute aortic syndrome (AAS) is a group of cardiovascular diseases that seriously threaten human life and health (1). With the improvement of living standards and the progress of aging, hypertension and various genetic diseases with connective tissue changes are the key points that increase the occurrence of AAS. In addition, the incidence rate of coronary atherosclerotic heart disease is also increasing. AAS is often concurrent with coronary artery disease, which should be paid more and more attention (Table 1). In recent years, with the progress of science and technology, a variety of CT angiography techniques have been applied in the clinic, greatly increasing the diagnostic rate of AAS combined with coronary heart disease (CAD) (2, 3). Because of the high mortality rate, AAS usually requires early diagnosis and immediate medical or surgical treatment. It is also very important for vascular and thoracic surgeons to evaluate the coronary artery condition of patients before surgery (Figure 1) because there is a direct relationship between various chronic diseases and CAD, which may have influence on the prognosis of patients. At present, the diagnosis of AAS mainly depends on imaging techniques (4–6). Except the traditional imaging techniques [CT angiography (CTA) and echocardiography], a variety of other techniques have also been developed, such as four-dimensional flow cardiovascular magnetic resonance (4D flow CMR) for evaluating the hemodynamic status of AAS, and positron emission computerized tomography and computer tomography (PET/CT), blood vessel CT and other techniques based on nanoparticles as a contrast agent for evaluating the inflammatory response of blood vessel wall. Due to the good sensitivity and specificity, aortic CTA is now the preferred imaging examination method for clinical diagnosis of AAS, and coronary CTA is one of the commonly used methods for CAD. The symptom of AAS combined with CAD and the pathology is anemia and bleeding, which is different from coronary artery stenosis ischemia requiring thrombolysis. AAS usually requires coagulation treatment, but not thrombolysis. Aortic CTA and coronary CTA should be identified for the diagnosis of diseases in clinic.

**TABLE 1:** Clinical need for aortic CT aortic angiography combined with CCTA disease.

Clinical need for aortic CTA combined with coronary artery CTA disease	Whether the emergency	Disease characteristics	Issues needing attention in radiology department
Aortic dissection	YES	A very urgent disease with a very high mortality rate	First time scan results as early as possible to prevent unexpected adverse events
Patients with aortic syndrome complicated with coronary heart disease	YES	There is a relative urgency to identify associated diseases	We need clear images to distinguish between patients’ diseases
Patients requiring coronary artery bypass grafting and Others	NO	We need information to prepare for surgery	Expensive equipment only available in big hospitals

## Acute aortic syndrome

Acute aortic syndrome includes acute aortic dissection (AD), intramural hematoma (IMH), and penetrating atherosclerotic ulcer (PAU). The most common clinical symptoms are severe pain and potential life-threatening aortic abnormalities. It is impossible to distinguish these aortic diseases through the symptoms or physical examination. Therefore, any clinically suspected related diseases should be further treated, including using non-invasive imaging techniques (5, 6). The prognosis of AAS is obviously related to the in-time diagnosis and appropriate treatment (6). Accurate imaging conclusion is able to change the natural history of AAS and improve the prognosis. The destruction of the aortic mesomembrane is a common feature of AAS. Patients diagnosed with AAS are accompanied by IMH or PAU, which is life-threatening and difficult to distinguish in the clinic. Severe pain is the most common symptom of AAS. Pain in part or other related symptoms reflect the initial destruction of the intimal layer and probably change along with the dissection extending along the aorta or involving other arteries or organs. AD is formed as follows: (a) the aortic intima and media are tearing by various causes; (b) blood enters the media through the tear, stripping the aortic intima and media under the high pressure of rapid cardiac contraction; (c) the aortic lumen is separated into false lumens. If not treated in time, the mortality rate of patients with AAS is high, 24 h for 33%, 48 h for 50%, 1 w for 75%, and 3-month for 90%, among which the mortality of patients with acute myocardial infarction caused by AD type A involving aortic valves and coronary arteries is higher (4, 7, 8). With the improvement of living standards and the progress of aging, hypertension and various genetic diseases with connective tissue changes are the main factors that increase AAS. The annual incidence rate of AD is about 2.8–6.0/100000. The results of the international AD registry showed that the average age of AD is 63 years old and 65% are men. However, the research results of AD in China showed an increasing trend and the average age of patients is about 51 years old, about 10 years old younger than that of European and American countries. In addition, the number of patients with CAD has also shown an obvious increasing trend (9), and its morbidity and mortality are getting higher and higher, rendering a great threat to the life and health of all mankind. Many AAS patients are complicated with CAD, and the degree of coronary artery stenosis also directly affects the surgical effect of patients (10, 11). Early diagnosis and intervention or treatment in-time can reduce the occurrence of CAD events during surgery, the mortality and economic burden of patients, and improve the quality of life of patients during the perioperative period.

## Imaging techniques of acute aortic syndrome

### Multi detector computed tomography (MDCT), transesophageal echocardiography (TEE), and magnetic resonance imaging (MRI)

In the diagnosis of AAS (12–15), chest radiography plays a very limited role, and catheter angiography is an invasive treatment method, which is not suitable for disease exclusion due to its high cost and low spatial resolution. MRI has a high definition and no radiation exposure, but it is not enough to be used in the diagnosis of acute AAS because of the extended scanning time and limited availability of acute assessment of the coronary artery. TEE has good diagnostic accuracy and is the basis of intraoperative aortic valve evaluation. However, it requires trained doctors and sedation, and its application in acute AAS is also limited. In acute AAS, TEE is basically applicable to unstable patients and patients with uncertain CTA. The basic objective of imaging examination is to confirm the diagnosis of aortic wall lesions and determination of the location, extension, and complications of the disease, and plan the most appropriate and in-time treatment methods. The critical advanced imaging technique for acute AAS is CTA because it has excellent diagnostic performance, wide availability, and fast and extensive diagnostic ability. If possible, CTA should perform cardiac gating technology to reduce possible artifacts. For patients without acute AAS and suspected of CAD or pulmonary embolism, coronary and/or pulmonary CTA should be considered to provide various imaging means. Therefore, the good versatility, fast acquisition, and high accuracy make MDCT the preferable choice for emergency settings. MDCT has become a valuable method for the diagnosis of AAS (16).

### Combination of aortic CT aortic angiography and coronary CT aortic angiography

Since the advent of CT in the early 1970s (17, 18), the scanning speed has always been the driving force for the development of CT technology along with the progress of various spiral CT technologies (Table 2). Spiral CT laid the foundation for the basic improvement of CT imaging in the early 1990s (19–21), especially for the faster volume coverage, multi-slice CT and multi-slice spiral CT, which further significantly improve the scanning speed and increase the use of common *z*-axis through the plane and better use the output of the X-ray tube. The significantly reduced inspection time (22–24) is very important in the clinic. In various cases, such as cardiovascular or aortic CTA, faster scanning speed helps to reduce intentional, unintentional and internal movement, and the amount of contrast agent for cardiovascular, and uncooperative patients, or pediatric (25). Although CT technologies have been developed fast, the diagnosis of AAS is increasingly dependent on aortic CTA in the clinic. Because of the complexity of the disease (accompanied by complications), a single aortic CTA cannot completely deal with the clinical problems (14, 26–29), for example, type I AD needs to perform as follows: (a) confirm whether there is an intimal film; (b) evaluate the degree of disease according to the aortic structure; (c) identify the true or false lumen; (d) judge the location of the breach; (e) identify whether the lesion is anterograde or retrograde; (f) identify whether the aortic valve orifice is incomplete, (g) confirm the degree and mechanism of incomplete closure; (h) identify whether there are cumulative branches and the degree of cumulative lesions. There are various AAS complicated with CAD, which results in the difficulty of clinical surgery. A clear diagnosis plays an important part in AAS. In the CTA report of the AD, emphasis should be given to evaluate the location and size of the primary intimal tear, the range and direction of involvement, and the location and size of the re-entry tear. Various descriptions should be involved (6, 15, 16): all branches and their patency, the origin of the rupture, the diameter of the aorta, the degree of thrombosis, the identification of the true and false lumens, and the amount of fluid around the aorta or pleural effusion. The coronary artery also needs to describe the extent of involvement and coronary artery stenosis, and whether there is effusion around the pericardium. The majority of the AD requires immediate surgical intervention, which is generally affected by the extent of type A dissection extending to the aortic root, the degree of involvement of the aortic valve, aortic arch, coronary artery, and large vessels. Furthermore, the high-risk characteristics of type B anatomy should also be described in the CTA report, including a variety of aspects: (1) the large entrance tear (>10 mm), (2) the location of the primary intimal tear and the measurement of the initial diameter of the aorta, (3) the expansion of the false lumen and the compression of the true lumen caused by the increase of the pressure in the lumen when partial false lumen thrombosis occurs, and (4) the poor perfusion of branches and organs caused by the collapse of the true lumen. Entire thrombosis of the false lumen is usually a good imaging finding and is associated with a good prognosis. The meridians of each part of the aorta and coronary artery also need to be described in the CTA report for the preparation of aortic surgery. Now more and more doctors choose to perform aortic CTA and coronary CT at the same time, but multi-CTA causes increased radiation and contrast agent doses for patients (30–33). With the developed technology, the technique of aortic CTA combined with coronary CTA has become a research focus for radiologists. It will be a hot topic about how to further reduce the scanning and contrast agent dose.

**TABLE 2:** Technical parameters of different combined aortic and coronary CT aortic angiography (CTA) methods.

Different CT machine types	Technical parameters	Scanning characteristics	Heart rate request	Whether the patient needs to cooperate with breathing	Image character	References
16&64	16&64 × 0.625 mm	Since two scans were required, two injections of iodine contrast medium were given. Therefore, both the radiation and iodine contrast agent dose is high.	usually < 75 bpm	Strict with breathing-hold	Aorta and coronary artery images were obtained by two different scans with appropriate heart rate	Shapiro et al.; Leschka; Ropers et al.; Yoshida et al. (34– 37)
256&320	256&320 × 0.625	Two scans were finished in one injection of iodine contrast medium, and saves contrast medium to the patient.	usually < 85 bpm	Need breath-holding	with 64-slice CT 320&256-slice CT provides significantly improved and more stable image quality at an equivalent effective radiation dose compared	Zhang et al.; Chen et al.; Li et al. (41– 43)
Dual-source CT	2 × 96&64 × 0.6 mm	Obtain the images of both aortic CTA and coronary CTA in one scan, which saves a lot of time and reduces the radiation dose to emergency patient.	generally no heart rate limit	Breathing-hold is not mandatory	The corresponding aorta and coronary artery images were obtained by one-time high-pitch scan	Bucher et al.; Karlo et al.; Carmelinda et al.; Wielandner et al. (44– 47)

#### Scanning technology of 64-MDCT

There are many ways to combine the aorta with the coronary artery CTA (CCTA). Various studies have shown that this kind of scanning is not suitable for being combined with CTA due to the limitation of scanning time of 64- and below-MDCT. The heart rate limitation of the coronary artery also brings objective difficulties to the scanning (34–37). However, this kind of scanning is still the most effective method to exclude AAS and CAD in grass-roots hospitals. Shapiro et al. (34) reported that the aortic CTA, coronary CTA, and even pulmonary CTA are able to be obtained by using the coronary artery procedure with a controlled heart rate. For the whole aorta, ECG gated CTA in patients with aortic disease, high radiation dose, and respiratory motion artifacts caused by long scanning time should be considered. Since CT examination accounts for about 2/3 of the total radiation dose of all radiation examinations, reducing the effective dose of CT examination will ultimately reduce the risk of medical-induced cancer incidence rate. The International Commission of Radiological Protection estimates that the additional life-time risk of fatal cancer in the entire population is about 1/10000 (per 200 sv). Some techniques, such as ECG gated dose modulation and reducing tube voltage, have been studied to reduce the high radiation dose of coronary CTA. However, due to the additional exposure at the beginning and end of spiral acquisition, the “over radiation” of ECG gated spiral technology increases the total radiation dose, even if there is ECG gated dose modulation. So, this method has obvious defects, such as large scanning dose and long breath-hold time. Compared with 256-MDCT, 320-MDCT and dual source CT, the imaging dose of 64-MDCT is significantly higher (38–40). However, this method is still an effective method for evaluating AAS and CAD in grass-roots hospitals.

#### Technical characteristics of 320 and 256-MDCT

320- and 256-MDCT are superior CT based on the wide body detectors (41–43). They have a 16 cm detector range and can obtain clear coronary artery images at one time scanning, which benefits to reduce the radiation and contrast agent dose in patients. The spiral technology is still needed for large-scale scanning, significantly reducing the time required for a one-time aorta scan. However, obvious cardiac pulsation artifacts still exist in this large-scale scan and cannot be completely eliminated, so sometimes it is necessary to scan the coronary CTA in AAS diagnosis. It has been proposed that a one-time contrast agent combined with ECG gated prospective scanning and rapid bed motion technology can significantly reduce the used amount of contrast agent and obtain clear aortic and coronary images.

Li et al. (43) reported that CTA by scanning the whole aorta and coronary artery (CA) using the prospective ECG gated wide volume scheme of 320-MDCT could provide useful information about the aortic CTA and coronary CTA with low radiation exposure. This method employed the advantages of wide detectors and a 10% exposure window in 320 row CTA with a single heartbeat, thus it would reduce the effective radiation dose compared with complete R-R exposure, and obtain diagnostic images in more than 95% of the coronary artery segments and the entire aorta and aortic root (sinus and valve). Although the patient’s radiation dose was obviously reduced compared with the full retrospective ECG gating technology, the effective dose was still high. In addition, this method significantly reduced the dosage of contrast agent and kidney damage in patients.

#### Dual source CT scanning technology

**FIGURE 2:**
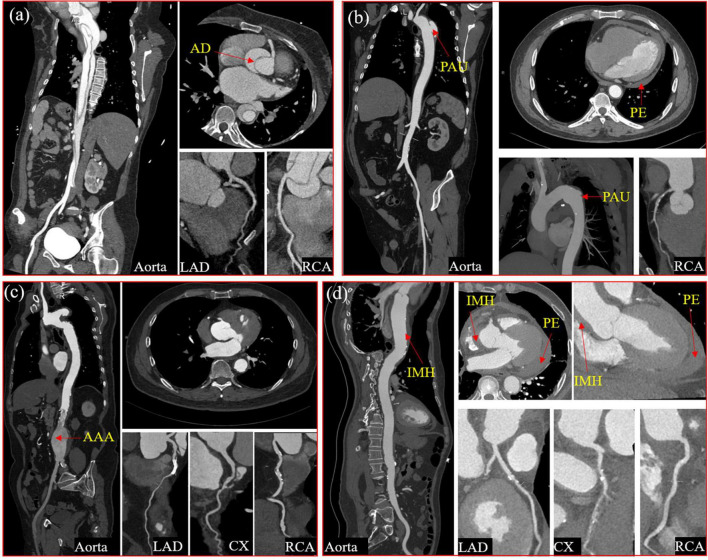
Four typical cases of aortic syndrome with coronary involvement. A case of Type I aortic dissection involved the ostium of the right coronary sinus with the significantly compressed right coronary artery **(a)** A case of penetrating aortic ulceration with intramural hematoma, pericardial effusion, and coronary atherosclerotic heart disease **(b)** A case of coronary atherosclerotic heart disease with abdominal aortic aneurysm after the right coronary artery stent implantation **(c)** A case of intramural hematoma with hemopericardium, compressed left and right coronary arteries, and coronary arteriosclerotic heart disease **(d)**. AD, aortic dissection; PAU, penetrating aortic ulcer; PE, pericardial effusion; AAA, abdominal aortic aneurysm; IMH, intramural hematoma; LAD, Left anterior descending coronary artery; CX, circumflex artery; RCA, right coronary artery.

The advent of Siemens dual source CT is a major technological improvement (25) because it allows to collect the data with abnormally high pitch and fast rotation speed and greatly reduces acquisition time and improves time resolution (Figure 2). For example, the ECG gated SEQ CTA technique can perform the whole coronary artery imaging (scan length 12–14 cm) in a single heart cycle with significantly reduced contrast agent and radiation dose. In addition, DS-CTA with or without ECG gating also provides high-quality images and slight motion artifacts in the whole aorta imaging (scan length: 50–80 cm) (44–47). Karlo et al. (45) proved that large pitch DS-CTA without ECG gating could provide a similar diagnostic image quality of the aortic valve-aortic root complex with ECG gating technology. Despite the advantages of obtaining short vascular segments (such as a coronary artery or thoracic aorta), the role of large pitch DS-CTA in whole thoracoabdominal aortic imaging is still a challenge and a controversial topic. Carmelinda et al. (44) reported that a large pitch aortic CTA scanning without ECG could provide enough information for the diseases like AD and had no motion artifacts in ascending the aorta like large pitch DS-CTA with ECG. They had no statistical difference in noise, signal-to-noise ratio, etc. Therefore, when evaluating the thoracic aorta, large pitch DS-CTA should be better than standard CTA, regardless of whether it is related to ECG. In addition, the double source DS-CTA with a large pitch reduced 72% of the radiation exposure, so it may be recommended for long-term monitoring of aortic disease. Wielandner et al. (47) reported that there is no significant difference in radiation dose between ECG automatically triggered large pitch CTA and non-triggered large pitch CTA. These two methods achieved a basic dose reduction of up to 86% compared with retrospective ECG gating. The median radiation dose assessed in their study is also significantly lower than that of prospectively triggered aortic CTA. With the advent of the third generation, dual source CT and various superior CTs, more and more combined large area CTA with low radiation and contrast agent dose will be used widely in the clinic. In conclusion, the technique of aortic CTA combined with coronary CTA offers accurate and reliable information for early detection, diagnosis and treatment, and post-operative follow-up. With the wide application of superior CT, double-low technology combined with iterative technology, three-dimensional reconstruction technology and the continuous progress of computer technology, the clinical mortality and misdiagnosis rate, and complications will be further reduced. Based on the fact that the current superior CT significantly reduces the radiation dose of patients, the ECG gated large pitch scanning mode is expected to become a new direction of imaging research on AAS.

#### CT post-processing technology

There are many methods for CT post-processing, mainly including multi planar reconstruction (MPR), shaded surface display (SSD), maximum intensity projection (MIP), volume rendering (VR), and CT virtual endoscopy (CTVE), etc.

### Contrast agent use in combination with CT aortic angiography

CT aortic angiography has become an imaging method for the diagnosis, follow-up, and preoperative planning of aortic disease (2–6). Due to the complexity of the disease, many patients with cardiovascular related diseases need to undergo combined coronary CTA (48–51). A large amount of radiation dose and acute renal injury remains the concern, especially in patients with aortic disease and cardiovascular disease, which usually require life-long imaging monitoring. The repeated CTA examination will bring an increased radiation dose and renal function damage to the patients. The recent guidelines indicate that the risk of acute renal injury is lower than the previously thought level. With the development of CT scan technology, such as high-power, high pitch protocol, or the newest iterative reconstruction algorithm, the potential savings of radiation and iodine dose of aortic CTA are increasing. In the past decade, the study achieved an average total iodine dose of 10.5–41 g for thoracic and abdominal aorta CTA and confirmed the diagnostic image quality of potential schemes (52–57). Optimizing the dose of radiation or iodine will reduce the potential risk for the patients.

Fink et al. (52) reported that the thoracoabdominal aorta CTA could be performed in non-obese and obese patients without high-level heart failure symptoms under the condition of very low radiation and iodine dose. At the same time, through the optimized CTA protocol adapted to BMI, the diagnostic image quality was guaranteed in 128-MDCT scanning technology. Wei et al. (53) believed that the aortic CTA technology with a double low imaging scheme (300 mg/ml, 1.5 ml/kg) and rate of 4–5 ml/s (low tube voltage and contrast agent concentration) significantly reduced the dose of radiation and iodine contrast agent while maintaining good image quality. Jia et al. (54) studied the feasibility of CCTA under low voltage on the third-generation dual source CT. They believed that the third-generation dual source CT could obtain high-quality images and high diagnostic accuracy for significant stenosis by using 70 kvp large pitch coronary CT angiography system, automatic tube voltage selection, and 30 ml low concentration contrast agent. However, this technique was not suitable for patients with high heart rate and serious coronary artery calcification because low kV photography was not sensitive to calcified plaques. Pan et al. (55) believed that compared with 100 kvp tube voltage combined with multi concentration contrast agent, 80 kvp combined with 270 mg/ml contrast agent was sufficient to ensure image quality, significantly reducing radiation and iodine intake dose, and the incidence of adverse reactions in the patients with low BMI and controlled HR. Zhang et al. (56) reported that it was feasible to perform the CCTA at 270 mg/ml and 100 kV. This low kV and contrast agent concentration combined with iterative reconstruction for CCTA imaging obtained the same image quality with that of conventional CCTA, significantly reducing the dose of radiation and iodine injection.

Although there are few reports about low kV and concentration of combined CTA, it is more and more used to reduce the radiation and contrast agent dose of patients in the clinic. Whether using BMI for the management of contrast agent or other methods to reduce the amount of contrast agent, it is feasible if it does not affect the image diagnosis quality.

## Summary and outlook

The occurrence of aortic disease complicated with coronary artery disease is increasing, and thus the technique of aortic CTA combined with coronary CTA is more and more applied in the clinic. However, a large amount of radiation dose and acute kidney injury are the main concern to patients, especially in grass-roots hospitals still primarily using 64- and 16-MDCT, although it is a practical diagnostic method in these hospitals. Therefore, it is also very important for clinical imaging doctors to further reduce the radiation and contrast agent dose of patients by using the current machines.

Although all kinds of superior CT are increasingly saving the radiation and contrast agent dose for patients, there is still room for technical choice and development. It will be still a challenge for imaging doctors to study how to further reduce the patient’s radiation and contrast agent dose by using 256- and 320-MDCT with wide body detectors.

More and more imaging doctors have begun to study how to realize one-time scanning in low-dose and large pitch to obtain clear aorta and CCTA images, especially after the use of Siemens dual source CT with large pitch. Although this scanning is not probable to completely replace angiography and other techniques, it is still effective in evaluating coronary artery stenosis rate in aortic related diseases. In the studies based on Siemens dual source CT, the radiation and contrast agent dose are obviously reduced by correlating ECG with large pitch scanning for AAS combined with CAD patients. However, there is no report about the personalized scanning scheme that accurately controls the time window of coronary artery scanning in this kind of technique. Therefore, extensive studies should be further performed to obtain clear images and bring more benefits to patients.

## Author contributions

HA collected and analyzed the data and drafted the manuscript. RX helped to analyze the data and revised the manuscript. YG reviewed the manuscript carefully, provided some suggestions and important pictures to the revised version, and gave the financial support. TW made substantial contributions to conception and design of the whole work, helped to acquire and analyze the data, and revised every version of the manuscript. All authors contributed to the article and approved the submitted version.
